# Sensitivity of Dielectric Properties to Wear Process on Carbon Nanofiber/High-Density Polyethylene Composites

**DOI:** 10.1007/s11671-010-9748-1

**Published:** 2010-08-21

**Authors:** Tian Liu, Weston Wood, Wei-Hong Zhong

**Affiliations:** 1 School of Mechanical and Materials Engineering, Washington State University, Pullman, WA 99164, USA

**Keywords:** Nanocomposite, Polyethylene, Carbon nanofiber, Wear resistance, Permittivity, Dielectric loss

## Abstract

We examined the correlation of wear effects with dielectric properties of carbon nanofibers (CNFs; untreated and organosilane-treated)-reinforced high-density polyethylene (HDPE) composites. Wear testing for the nanocomposites over up to 120 h was carried out, and then, dielectric permittivity and dielectric loss factor of the polymer composites with the increased wear time were studied. Scanning electron microscope and optical microscope observations were made to analyze the microstructure features of the nanocomposites. The results reveal that there exist approximate linear relationships of permittivity with wear coefficient for the nanocomposites. Composites containing silanized CNFs with the sufficiently thick coating exhibited high wear resistance. The change in permittivity was more sensitive to the increased wear coefficient for the nanocomposites with lower wear resistance. This work provides potential for further research on the application of dielectric signals to detect the effects of wear process on lifetime of polymeric materials.

## Introduction

Making polymers into nanocomposites with the addition of appropriate nanofillers has been shown to be an effective way to obtain a multitude of enhanced properties and even extend to multi-functionalities not normally considered possible for polymeric materials. A variety of properties, such as physical, thermal, mechanical and others, as well as combinations of them, have been investigated for numerous nanocomposites in order to meet the burgeoning demands of industry. For example, carbon nanofillers, such as carbon nanotubes (CNTs) and carbon nanofibers (CNFs), not only improve electrical conductivity and dielectric properties, but also show enhanced tribological performance for polymers [1-8]. In addition, it has been widely reported in many studies that effective treatments for these nanofillers, including purifications, and/or chemical or non-chemical functionalization, are necessary in order to develop high-performance nanocomposites [9-13].

Among various nanoscale fillers, owing to high aspect ratios and potential for multi-functionality, CNTs and CNFs are commonly used as the reinforced materials for polymer composites [14-17]. For bulk and large volume inexpensive polymers, CNFs are attractive due to the excellent stability and quality of the commercial products. For example, the purity of Pyrograf^®^ nanofibers, which have been widely used, is >98%, and thus, the use of CNFs will not require time/energy-consuming purification processes. In addition, CNFs have graphene-layered structures, which provide vast numbers of active edges on the surface for functionalization purposes. The enormous amount of edges on CNFs enable these nanofillers to be functionalized easily compared to CNTs, which have stable and smooth tube structures, requiring much more dramatic methods of functionalization, typically using strong acids [12]. Therefore, not only the low cost of the raw CNF materials, but also the easy treatments of the nanofibers make this type of nanofiller more attractive and practical for bulk nanocomposite manufacturing and industrial applications.

Numerous attempts of using functionalized CNFs as the reinforcement in nanocomposites have been investigated to accomplish the purpose of improving interaction and adhesion between the nanofillers and polymers [18-24]. For composite systems with non-polar polymer matrices, to dramatically improve the interactions between nanofibers and non-polar polymer matrices is challenging due to limited and weak van der Waals forces. It has been reported that much stronger physical entanglement interactions can be realized through *thicker* coating layers on the nanofibers that function as bridges, or transition zones, to enhance entanglements between the nanofibers and non-polar polymers, e.g. polyethylene [15-17]. In our previous studies, we successfully synthesized silanized CNFs with thick silane coatings (~46 nm) [25]. Dynamic mechanical analysis and wear-testing results indicated that CNFs treated with such thick silane coatings are effective nanofillers for enhancing mechanical and tribological properties for polyethylene. Our current studies suggested a great potential of this type of silanized CNFs in improving wear resistance and mechanical properties for high-performance polyethylene, i.e. ultra-high molecular polyethylene, so that the resulting nanocomposites will be conceivably be attractive for applications to joint replacement systems with lifetimes significantly extended over the pure polyethylene material [26-30].

However, there is dearth of information on how mechanical behavior such as wear/friction processes (during the usage of the artificial joint) affects structures and properties of nanocomposites used in biosystems. Therefore, there are many concerns that need to be addressed on the application of nanomodified polymers to joint replacements. One of the most critical issues is related to the effects of the wear process on the stability of nanocomposites, both physically and chemically. Even though in the resulting nanocomposites, silanization provides non-polar coatings on the nanofiber surface and polyethylene is also a non-polar polymer, it is not known whether the continuous wear process can create polar groups in the nanocomposites and what relationship would be between the wear rate/wear period and the potential polar group created by the wear process. Such polar groups inside the polymer matrix may directly influence the chemical stability of the polymer and thus affect the practical lifetime of the joint replacements. Moreover, if the wear process creates polar groups in the polymer matrix, such groups can also be maintained in the debris, which has been proved to have a relationship with bone loss.

It is well known that dielectric response is a reflection of dipole movements in insulative materials under an electric field. There are many applications of dielectric measurements to polymeric materials, since dielectric response is very sensitive to polar groups in the polymers [31,32]. For example, more than 25 years ago, there were studies on in situ monitoring of epoxy curing through continuous measurement of dielectric performance during curing processes [33-36], since with the increase in degree of cure, the molecular weight increases continuously, which has a direct impact on the movement of polar groups within the epoxy. In recent years, there have been many studies reporting on the effects of nanofillers, such as CNFs and CNTs, on the dielectric properties of various polymers including polyethylene [37,38]. However, there has not been any reported investigation into the relationship between effects of a wear process on polymers and the dielectric characteristics.

In this study, we have investigated the dielectric property changes with wear time and the degree of wear for HDPE nanocomposites containing our previously silane-treated CNFs (thin and thick coatings). The silane coatings not only enhanced the interaction of HDPE matrix and the CNF reinforcement, but also provided a non-polar hydrocarbon layer to cover the nanofiber surface. We hypothesized that the wear process can create polar groups by cutting some polymer chains and causing separation of nanofibers with the polymer matrix, as well as causing damage to the nanofiber structure. Thus, it may be deduced that with the longer wear time, dielectric response can be stronger since more wear can result in more polar groups on the surface and near the surface (since wear on the micro scale is not uniform and successive wear broadens and fills in the affected area, asymptotically approaching totality). In addition, we postulate that the coating thickness can not only affect wear resistance of the resulting composites, but also impact the dielectric properties of the composites. In order to compare with a nanocomposite containing pre-existing polar groups, oxidized nanofibers (ox-CNFs) were used for this purpose. Subsequently, we prepared four types of nanocomposites including three types of composites with different thickness of silane coatings on the CNF surface. In order to gain both sufficient sensitivity of dielectric responses and relatively preferable wear resistance, a 3% wt concentration of carbon fibers was chosen. The experimental results remarkably revealed that approximate linear relationships of permittivity with wear coefficient for the nanocomposites. The change in permittivity was more sensitive to the increased wear coefficient for the nanocomposites with lower wear resistance.

## Experimental Procedures

### Sample Preparation

The high-density polyethylene used as a matrix in this research was supplied by Equistar (LB010000) with density of 0.953 g cm^-3^. The pretreated ox-CNFs as the filler were obtained from Applied Sciences Inc., which are approximately 60 to 150 nm in diameter and 30 to 100 microns in length. Octadecyltrimethoxysilane (ODMS) (90% technical grade) was manufactured by Sigma–Aldrich. Acetone was obtained from J.T. Baker. Ethanol was purchased from Decon Laboratories Inc.

The ox-CNFs were modified under subsequent treatment in boiling ODMS–ethanol solution. Then, the condensation reaction of ox-CNFs and silane coupling agent can occur due to the reactive hydroxyl groups on the surface of organosilane after hydrolysis, forming a silane layer to cover ox-CNF surface. By changing the ratio of ODMS to ox-CNF added and the percentage of ethanol and water in this reaction, we can control the degree of hydrolysis and the thickness of silane coating. In the previous work, three calculated coating thicknesses were applied using TGA data, which is about 1.2 nm for silanized CNF-A, 2.8 nm for silanized CNF-B and 46 nm for silanized CNF-C [25].

The concentration of 3 wt% CNFs for both ox-CNFs (Nanocomposite-ox) and silanized CNFs (Nanocomposite-A with the thinnest coating, Nanocomposite-B with medium coating thickness and Nanocomposite-C with the thickest coating) were mixed with HDPE by a Haake Torque Rheometer for uniform dispersion. Mixing was set at 170°C with a rotator speed of 30 rpm. The order of adding the materials was as follows: half amount of HDPE, CNFs, and then another half amount of HDPE. The speed was then raised to 70 rpm for 15 min.

The polymeric nanocomposites were hot-pressing at 178°C for 10 min via a hydraulic presser. Then, they were allowed to cool down to room temperature naturally after turning off the heat. All samples were cut for wear testing and dielectric testing with the same size of 20 mm × 20 mm and similar average thickness around 2.5 mm.

### Wear Testing

**Figure 1:**
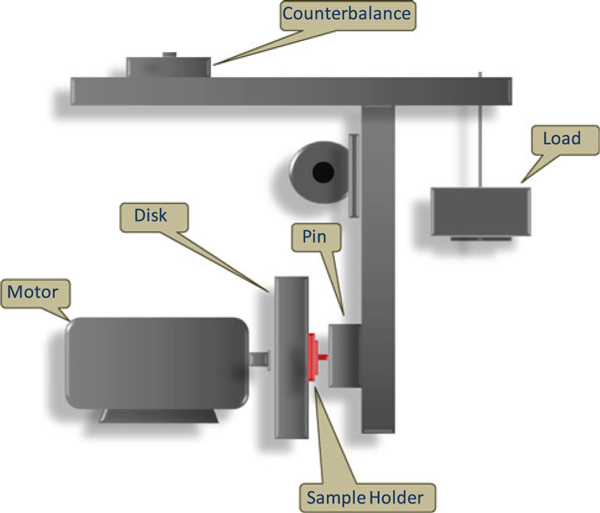
**Schematic of pin-on-disk wear-testing apparatus**.

The detailed study of friction and wear properties of the developed composite was performed in a custom-built rig for the samples with a vertical 1020 carbon steel disk, which is rich in iron and carbon. (HITACHI L200 Series, supplied by Hitachi Industrial Equipment system Co., Ltd). The components of the equipment are shown in Figure 1. The effective radius of the disk was 65 mm, and testing was performed at 180 rpm. Also, a normal force of 36 N was applied to the sample specimen with the friction surface of 20 mm × 20 mm. All the tests were carried out at room temperature. For each sample, the weight change and wear coefficient were calculated and recorded after every testing interval of 24, 48, 72, 96 and 120 h. In order to avoid the influence of previous tests, the disk was cleaned thoroughly with acetone and dried and then polished by fine sand paper of the type silicon carbide 220b (220 grit) prior to further use.

Specific wear coefficient was calculated using the following equation:

(1) $$w=\frac{\Delta m}{\rho Fd}$$

where *w* is the wear coefficient, △*m* is the weight loss, *ρ* is the density of composites, *F* is the normal force and *d* is the linear sliding distance. An Adventurer Pro analytic scale was used to determine mass loss, with a detectable range of 0.1 mg.

### Dielectric Testing

The frequency dependence of permittivity and dielectric loss under a constant temperature was determined by using an Alpha-N High Resolution Dielectric Analyzer equipped with Au parallel plate sensors, with frequencies from 10^-3^ to 10^7^ Hz. Debris produced by friction was removed by nitrogen gas rather than a solvent to avoid any effects related to them, and then dielectric analysis testing was conducted. The change in dielectric properties was measured before and after each wear testing (24, 48, 72, 96 and 120 h, respectively). The frequency ranges were chosen from 10^1^ to 10^6^ Hz for both dielectric constant and loss factor.

### Characterizations

The microstructure features on the fracture interface of both ox-CNF and silanized CNF nanocomposite specimens were observed by field-emission scanning electron microscopy (FESEM type Quanta 200F) in order to characterize the interaction and adhesion between fibers and matrix. FESEM images for fractured surfaces were prepared by freezing in liquid nitrogen for 10 min prior to fracturing. The surfaces of all samples were sputter-coated with gold for electrical conductivity. Additionally, the morphology of the composites was observed using optical microscope (Olympus BX51TRF) equipped with a camera (Olympus U-CMAD 3).

## Results and Discussion

### Morphology and Wear Resistance

**Figure 2:**
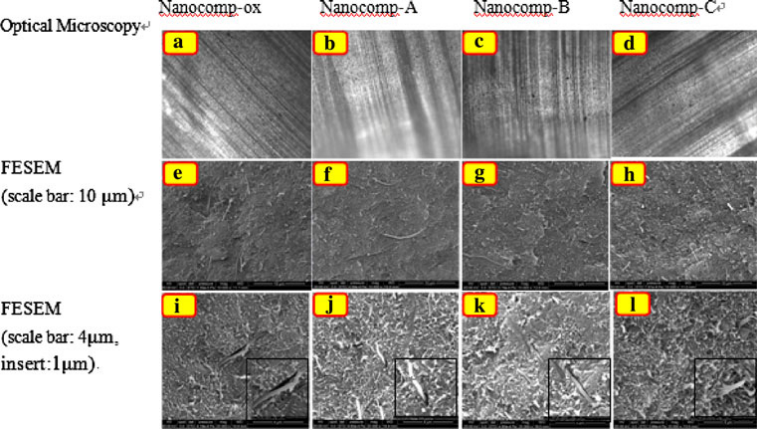
**Optical microscopy images (1st *row*), FESEM micrographs of surfaces of HDPE nanocomposite specimens for wear testing: reinforced by ox-CNFs: a, e, i; silanized CNFs with 1.2-nm coating thickness: b, f, j; silanized CNFs with 2.8-nm coating thickness: c, g, k; silanized CNFs with 46-nm coating thickness: d, h, l**.

Figure 2 shows optical microscopy and FESEM with different magnification images of the specimen surfaces of the nanocomposites for wear testing. From (a), (b), (c) and (d), it can be seen that those four types of composites show similar smoothness of the surface; only very few small CNF agglomerates (small black spots) were found in the samples, suggesting that the nanofibers were well distributed in the HDPE matrix by means of the melt mixing process.

Dispersion of the various CNFs (oxidized and silanized) in the HDPE matrix was observed through field-emission SEM (FESEM). The FESEM images in Figure 2e–l are typical fracture morphology of the nanocomposites before wear testing. It can be seen that there are no obvious agglomerates of nanofibers found in the composites, indicating dispersion and distribution of the nanofibers in the composites were uniform. For the nanocomposites with ox-CNFs, Nanocomp-ox, there was some fiber pullout (Figure 2e, i). Also, there were gaps between the nanofibers, and the matrix revealing the adhesion was poor. For the nanocomposites reinforced by silanized CNFs, Nanocomp-A (Figure 2f, j) and Nanocomp-B (Figure 2g, k), which have thinner coating layers on the CNF surface (1.2 nm and 2.8 nm, respectively), there were also fibers pulled out, especially for Nanocomp-A with the thinnest coating thickness. This phenomenon indicates that the poorer interaction and adhesion between nanofiller and HDPE due to a very thin coating likely result in insufficient entanglement of nanofibers with the polymer chains. The loose entanglement from the thin silane coatings cannot induce an increased strength between the fiber coating and matrix so that the fibers are easier to be pulled out. For Nanocomp-C (Figure 2h, l), there were no obvious long fibers exposed on the surface of matrix and few were pulled out, indicating good adhesion and interaction were obtained from Nanocomp-C with the thick coating. Also, there were few voids between the two phases. This clearly suggested that a thicker silane coating may allow for hydrophobic polymer chains to entangle onto the coating, thus attributing to an improved adhesion and interaction between the modified CNFs and HDPE matrix. Therefore, the higher wear coefficients were appeared from the nanocomposites with the lightly silane-treated CNFs (with thin coatings), especially for Nanocomp-A with the thinnest coating layer on the CNF surface, as revealed in below results.

**Figure 3:**
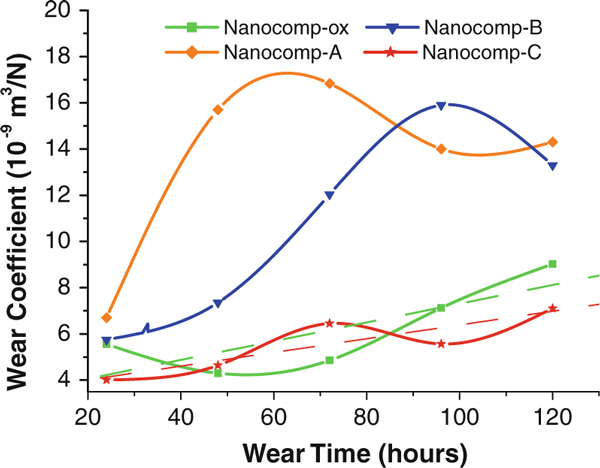
**Comparison of wear coefficient and wear time for all specimens**.

The effect of the silane-coating thickness on CNFs on the wear coefficient of the resulting nanocomposites versus wear time was studied. The wear coefficient comparison of composites was calculated by Eq. 1. It can be seen from Figure 3 that the tendency of wear coefficients increased with wear hours for all the nanocomposite samples. The change rates (slope of the curves) of the lightly silanized CNFs (with 1.2-nm and 2.8-nm coatings)-reinforced HDPE composites, Nanocomp-A and Nanocomp-B, were much higher than those of oxidized CNFs, Nanocomp-ox, and heavily silanized CNFs (with 46 nm coating)-reinforced composites, Nanocomp-C. For Nanocomp-ox, the change rate of wear coefficient was lower than those of Nanocomp-A and Nanocomp-B. This may be owing to the high stiffness of the surface of the ox-CNFs in comparison with the silane coatings on the CNF surface. For the nanocomposites with thin coating layers on the CNF surface, they showed poor wear resistance, even lower than Nanocomp-ox, in particular for Nanocomp-A. Possibly, this was caused by the insufficient physical entanglement between coating and matrix, and thus, the thinner coatings could not provide strong interfacial interaction between the nanofibers and the matrix in the composites. In comparison, Nanocomp-C displayed a minimum slope, reflecting the best wear resistance. Consequently, the results indicate the thick silane coating improved wear resistance of the composites system effectively.

### Permittivity Analysis

**Figure 4:**
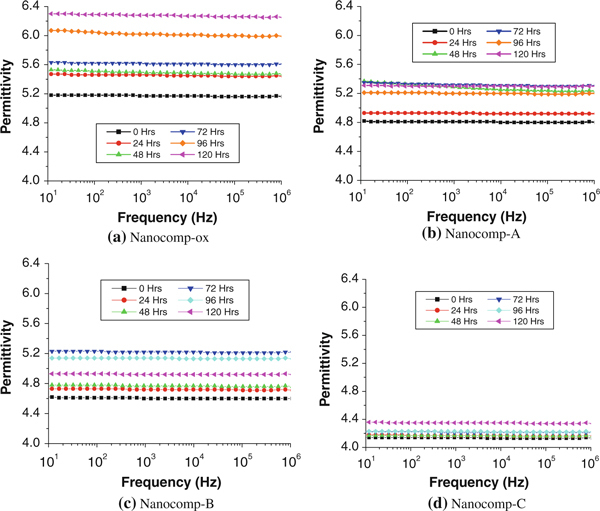
**Frequency dependence of permittivity with increased wear time for oxidized and silanized CNFs with different fiber coating thicknesses in HDPE nanocomposites**.

The variation in permittivity with the frequency over a range from 10 to 10^6^ Hz at different wear times for the four nanocomposites is presented in Figure 4a–d. It can be seen that the real-part permittivity (in short, permittivity) values of all the composite samples were independent of frequency before and after wear testing, owing to the concentration of CNFs (3 wt%) in the composites being lower than the percolation threshold [39-42]. Additionally, the effect of ox-CNF on the dielectric constant of composites was more influential [32] than the that of silanized CNFs, i.e. silane treatment for the nanofibers decreased the dielectric constant of the resulting composites noticeably. Also, with the increase in coating thickness on the CNFs in the composites, permittivity showed a lower sensitivity to the wear process. This occurred due to the change resulting from the condensation reaction between silanol and hydroxyl groups, thereby reducing the polar groups on the surface of the CNFs and then forming the long non-polar hydrocarbon chains onto them.

**Figure 5:**
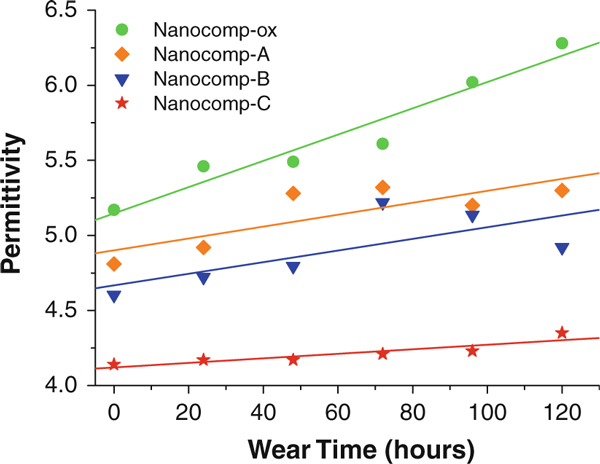
**Summary of permittivity-changing trends at 1 × 10^3^ Hz with increased wear hours of the four samples (The slopes of these curves were presented in Table 1)**.

Owing to permittivity was independent on the frequency before and after wear testing, we chose a certain frequency level to make a comparison of the four types of composite samples. From Figure 5, the change trends of permittivity at the frequency of 1 × 10^3^ Hz over the increasing wear time were obtained. It displayed the effect of the wear procedure on the dielectric constant of both ox-CNF/HDPE and silanized CNF/HDPE systems. The tendency of all samples was increasing with wear. For ox-CNF-reinforced composites, the hydroxyl groups on the fiber surface were the main contribution of the increased dielectric constant. For silane-treated CNF composites, although the changes were not as obvious as ox-CNF/HDPE because of the silane coating acting as a protective covering that leads to less polar groups in the molecular structure, the trends were still increasing. We supposed that the wear process can create polar groups by cutting some polymer chains and causing separation of nanofibers with the polymer matrix, as well as causing damage to the nanofiber structure. Thus, it may be deduced that with the longer wear time, dielectric response can be stronger since more wear can result in more polar groups on the surface and near the surface. Additionally, the broken chains or polar groups (after the wear process) can be affected by the external electrical field acting on the composite samples. The contribution to the dielectric results may have been due to the active groups produced on the worn surface so causing the variation of polarization. It may come from the slight changes of molecular structure in the composites and/or the active groups were produced on the worn surface so causing the variation of polarization. The relationship between coating thickness on the CNF surface and the permittivity of the resulting nanocomposites, as well as their change rates (slope of the lines shown in Figure 5) becomes clearer in Table 1. It can be noted that the change rate of permittivity over wear time was decreased with the increased coating thickness by the comparison of the slope. The slope of Nanocomp-C containing the thickest coating on the CNFs (1.51) was 82.7% lower than Nanocomp-ox (8.75), 61.9% lower than Nanocomp-A (3.96) and 61.1% lower than Nanocomp-B (3.88). It indicated that the thicker coating has the ability to reduce the change rate of permittivity versus wear time. This may be due to the fact that much more silanol groups react by an increased rate of hydrolysis, and thus a higher density of crosslinking network of silane coating to be formed so that diminishing the hydroxyl groups on CNFs of the composites significantly. Therefore, Nanocomp-C with the thickest silane-coating filler exhibited the lowest permittivity and the lowest increasing rate of permittivity over wear time, and Nanocomp-A with the thinnest filler coating thickness exhibited the highest initial permittivity and highest rate of increase. Figure 5 shows that increasing the coating thickness from zero to maximum have a stultifying effect on the influence of wear time (and wear rate) on permittivity.

**Table 1:** Effect of silane-coating thickness of CNFs on permittivity of the nanocomposites before and after wear testing

Samples	Thickness of silane coating on CNF surface (nm)	Permittivity before wearing	Permittivity after 120-h wearing	Slope (from Fig. 4) × 10^-3^
Nanocomp-ox	0	5.2	6.3	8.75
Nanocomp-A	1.2	4.8	5.3	3.96
Nanocomp-B	2.8	4.6	4.9	3.88
Nanocomp-C	46	4.1	4.4	1.51

### Permittivity Versus wear Coefficient

**Figure 6:**
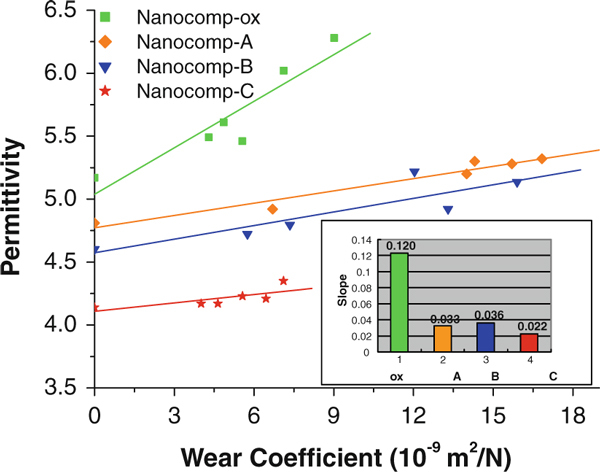
**Permittivity-changing trends at 1 × 10^3^ Hz versus corresponding wear coefficient of the ox-CNF/HDPE with untreated and treated ox-CNFs**.

In order to discern more clearly the effect on permittivity due to the wear process, the relationship between permittivity and the corresponding wear coefficient was developed in Figure 6 from the relationships in Figures 3 and 5, eliminating the time variable and making it more generally and directly related to the degree of wear. It can be seen that there exists an obvious relationship between the permittivity and the wear coefficient. The permittivity increased linearly with the increase in wear coefficient. By comparison of the slopes of the lines, Nanocomp-ox exhibited a much higher rate of permittivity increase than the composites with silane-treated CNFs. It demonstrated that the silane treatment induced a decreasing presence of polar groups on the surface of the carbon fibers; accordingly, fewer reactive groups were influenced by the external electric field. Wear effects on silanized CNF-reinforced HDPE composites were consistently less sensitive to the permittivity change driven by the wear process for all silane-based materials. Among those, the trend is clear that increasing the coating thickness caused a reduction in sensitivity with Nanocomp-C with the thickest coating on the CNFs, with the smallest slope. This is due to a relatively complete hydrolysis leading to much less hydroxyl groups inside. Conversely, the change in permittivity was more sensitive to the increased wear coefficient as the coating thickness of fillers in the composites approached zero.

### Loss Factor Analysis

**Figure 7:**
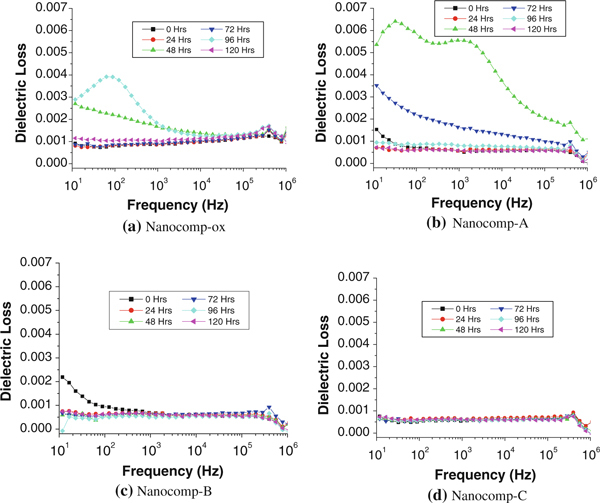
**Frequency dependence of dielectric loss over wear time for HDPE nanocomposites with ox-CNFs and silanized CNFs with different coating thickness, respectively**.

Figure 7a–d exhibited a variation in dielectric loss factor with frequency as wear time increases. It can be noted that Nanocomp-ox and Nanocomp-A showed signs of fluctuation after wearing, especially for Nanocomp-A. For Nanocomp-ox, the main reason for that fluctuation was the amount of hydroxyl groups on the fiber surface, being more sensitive to the frequencies. For Nanocomp-A, besides the influence of residual hydroxyl groups, after silane treatment, we suspect that the thin coating layer may be easily damaged, and thus, there were more polar groups created by the wear process owing to the thin coating layer on the fiber surface. Therefore, the fluctuation was shown to be greater than in Nanocomp-ox. In comparison, Nanocomp-B presented little fluctuation and Nanocomp-C showed effectively constant dielectric loss versus frequencies over wear time; the amplitude of fluctuation of the curves was smaller over frequency for the nanocomposites with the increased coating thickness on fiber surface. Thus, it indicated, again, for the Nanocomp-C, the thicker silane coating could protect the CNFs better, even up to the longest wear time, i.e. 120 h. The dielectric loss was still stable throughout the frequency range. In the other words, a sufficient coating layer surrounding the nanofiber surface is efficient for providing good protection from the long-period wear process through generating much less polar groups. This result suggests that CNF/HDPE nanocomposite stability during a longer wear process can be realized by providing sufficient coating thickness to the CNFs, and potentially the lifetime of the nanocomposites experiencing the wear process may be extended significantly.

To postulate from these results further, the debris generated from nanocomposites with the thicker silane coatings on the CNFs may also have the lowest amount of polarized groups/segments, which will be extremely positive for the application of the polymeric materials to bone joint replacement.

**Figure 8:**
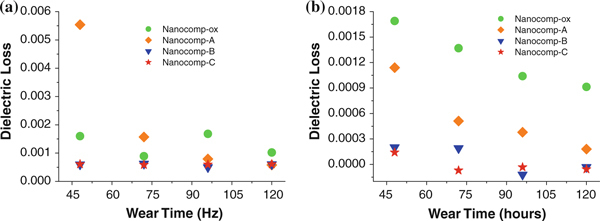
**Dielectric loss versus wear hours at a 1 × 10^3^ Hz and b 1 × 10^6^ Hz for nanocomposites**.

Based on the above testing results, the values of dielectric loss of the four composites were compared by choosing two frequency levels (10^3^ and 10^6^ Hz), which are shown in Figure 8. Due to the existence of instability factors before 48 h of wear time (Figure 7), we only chose four wear periods (48, 72, 96 and 120 h, respectively) for comparison. For the lower frequency level (10^3^ Hz, Figure 8a), the dielectric loss is low for almost all composite samples over the wear time up to 120 h (below 0.002), except Nanocomp-A after 48-h wear. In comparison, the high frequency level (10^6^ Hz, Figure 8b) can reflect the responses of smaller polar groups than those under the low frequency level. Under this high frequency level, Nanocomp-ox shows the highest dielectric loss, and Nanocomp-A with thinnest silane coating on CNF surface has the second highest loss values, which implies that the number of the smaller polar groups is large. It is clear that the silanized CNF-reinforced HDPE samples had much lower dielectric loss than oxidized CNFs/HDPE samples, and with the thicker silane coating on the nanofiber surface, the resulting nanocomposites, such as Nanocomp-B and Nanocomp-C, show lower loss results, which implies that they possess fewer polar groups. This result pointed out the decreasing of the wear-generated polar groups for nanocomposites can be realized by synthesizing thicker coatings onto the filler surface of the composites. Thus, it was concluded that sufficient silane coatings can efficiently contribute to a reduction in dielectric loss in such nanocomposites.

## Conclusions

Since there has never been any reported study indicating the effects of a mechanical process, such as wear, on dielectric properties of polymeric materials, this original study is purely an exploratory work. The results reveal that there exist relationships between wear effects and dielectric properties for polymeric nanocomposites. Composites containing silanized CNFs with sufficiently thick coatings exhibited high wear resistance. The permittivity of the nanocomposites decreased obviously for the nanocomposites with appropriate silane treatment for the nanofillers throughout the 120-h wear process. For all the nanocomposites prepared in this study, both ox-CNF/HDPE and silanized CNF/HDPE nanocomposites, dielectric constants were sensitive to the wear effects. It was revealed that approximate linear relationships of permittivity with wear coefficient existed for the nanocomposites. Also, the change in permittivity was more sensitive to the increased wear coefficient for the nanocomposites with lower wear resistance. Additionally, the silane-coating thickness played a significant role on the resistance of dielectric loss for the nanocomposites.

This work provides potential for the in-depth research on the application of dielectric signals to detect the effects of wear process on lifetime of polymeric materials for the application to joint replacement systems. It may also be extended to the more general case of detecting damage to structural composites in many applications.
